# Evaluation of a new developed disposable and portable bronchoscopy system

**DOI:** 10.1186/s12890-022-01933-z

**Published:** 2022-04-08

**Authors:** Zhixin Liang, Guanzhou Zhou, Yi Li, Fei Pan, Jiaqi Zeng, Zhe Luan, Qiang Zhu, Yi Xu, Nana Zhang, Lei Xiang, Yunxiao Jia, Gang Sun, Yunsheng Yang

**Affiliations:** 1 Department of Pulmonary and Critical Care Medicine, The First Medical Center, Chinese PLA General Hospital, Beijing, 100853 China; 2 Institute of Digestive Diseases, The First Medical Center, Chinese PLA General Hospital, Beijing, 100853 China; 3 Medical College of Nankai University, Tianjin, 300071 China; 4 Daichuan Medical (Shenzhen) Co., Ltd., Guangdong, 518000 China; 5 Laboratory Animal Center, Chinese PLA General Hospital, Beijing, China; 6 National Clinical Research Center for Geriatric Diseases, Chinese PLA General Hospital, Beijing, China

**Keywords:** COVID-19, Coronavirus disease, Bronchoscopy, Disposable bronchoscope, Infection

## Abstract

**Background:**

Bronchoscopy is critical in the treatment of patients with coronavirus disease (COVID-19), and its use is associated with the challenges of stringent sterilization and virus transmission risk. We developed a disposable and portable bronchoscope (YunSendo-R) and compared its safety and function with those of current reusable and single-use bronchoscopes using an animal model.

**Methods:**

We compared the YunSendo-R system with a commercially available reusable bronchoscope (Olympus, BF-H290) and single-use bronchoscope (Ambu, Ambu® aScope3™). Eight physicians used the three types of bronchoscopes to operate on Guangxi Bama mini pigs. Each operator performed bronchoscopy and completed a 10-point Likert scale questionnaire for evaluating visual ability and manoeuvrability. Operation time and scores were collected.

**Results:**

Operation time had no significant differences among the three bronchoscopes. In visual ability, the YunSendo-R bronchoscope showed superior performance to the Ambu bronchoscope in image clarity, colour contrast, and illumination (*P* < 0.05) and no significant difference in performance compared with the Olympus bronchoscope (*P* > 0.05). The YunSendo-R bronchoscope had similar manoeuvrability to the Olympus bronchoscope and better scope tip flexibility than the Ambu bronchoscope (*P* > 0.05). No relevant complications were reported.

**Conclusion:**

We have developed a new bronchoscopy system with the advantages of disposability and portability, which was effective and safe in an animal model. It has better visual ability than the Ambu bronchoscope and similar visual ability and manoeuvrability to the Olympus bronchoscope. The YunSendo-R bronchoscope is a promising device for clinical practice, especially in reusable-endoscope-transmitted infectious diseases such as COVID-19.

## Background

The currently raging infectious coronavirus disease (COVID-19) caused by the severe acute respiratory syndrome coronavirus 2 (SARS-CoV-2) mainly presents with fever and respiratory symptoms. Patients with severe infection tend to develop hypoxia and dyspnoea, progressing to acute respiratory distress syndrome. Bronchoscopy has become essential in the treatment of COVID-19 patients; however, direct contact with reusable bronchoscopes in reprocessing, especially during aerosol-generating procedures, has a risk of virus transmission [1–3]. Additionally, even with advanced and strict endoscope cleaning systems and procedures, real-world high-level disinfection is still an imperfect process, with a report showing microbial growth in 58% fully reprocessed bronchoscopes, including mould, *Stenotrophomonas maltophilia*, *Escherichia coli*, and *Shigella* species [4]. Reusing the bronchoscopes may involve insufficient cleaning, disinfection, or decontamination, and result in cross-infection, mostly by *Pseudomonas aeruginosa* and mycobacteria, which affects patient health and increases medical expenses [5, 6].

Disposable endoscopes are beneficial equipment as they eliminate complicated sterilization procedures, saving cost on reagents and staff. Furthermore, single-use bronchoscopes minimise the risk of cross-infection and virus transmission among both patients and staff. Their use has been widely recommended by multiple associations and guidelines during the COVID-19 pandemic [7, 8].

However, conventional bronchoscopy systems tend to be equipped with a heavy host, which has to be used in a specific room, thereby making them unavailable in settings such as emergency rooms and intensive care units. Patients in such settings have no access to immediate airway interventions. Portable system with ready accessibility can be used at a patient’s bedside in case of a serious condition, facilitating the use of urgent bronchoscopy when required [9].

Our team has developed a portable endoscopic system with a single-use gastrointestinal endoscope and used it for patients with digestive tract bleeding associated with COVID-19 [10, 11]. Here, we report the new disposable bronchoscope with a portable system (YunSendo-R system).

## Methods

This study was approved by the Institutional Animal Care & Use Committee (IACUC) of Chinese PLA General Hospital with number 2020-X16-36.

**Fig. 1:**
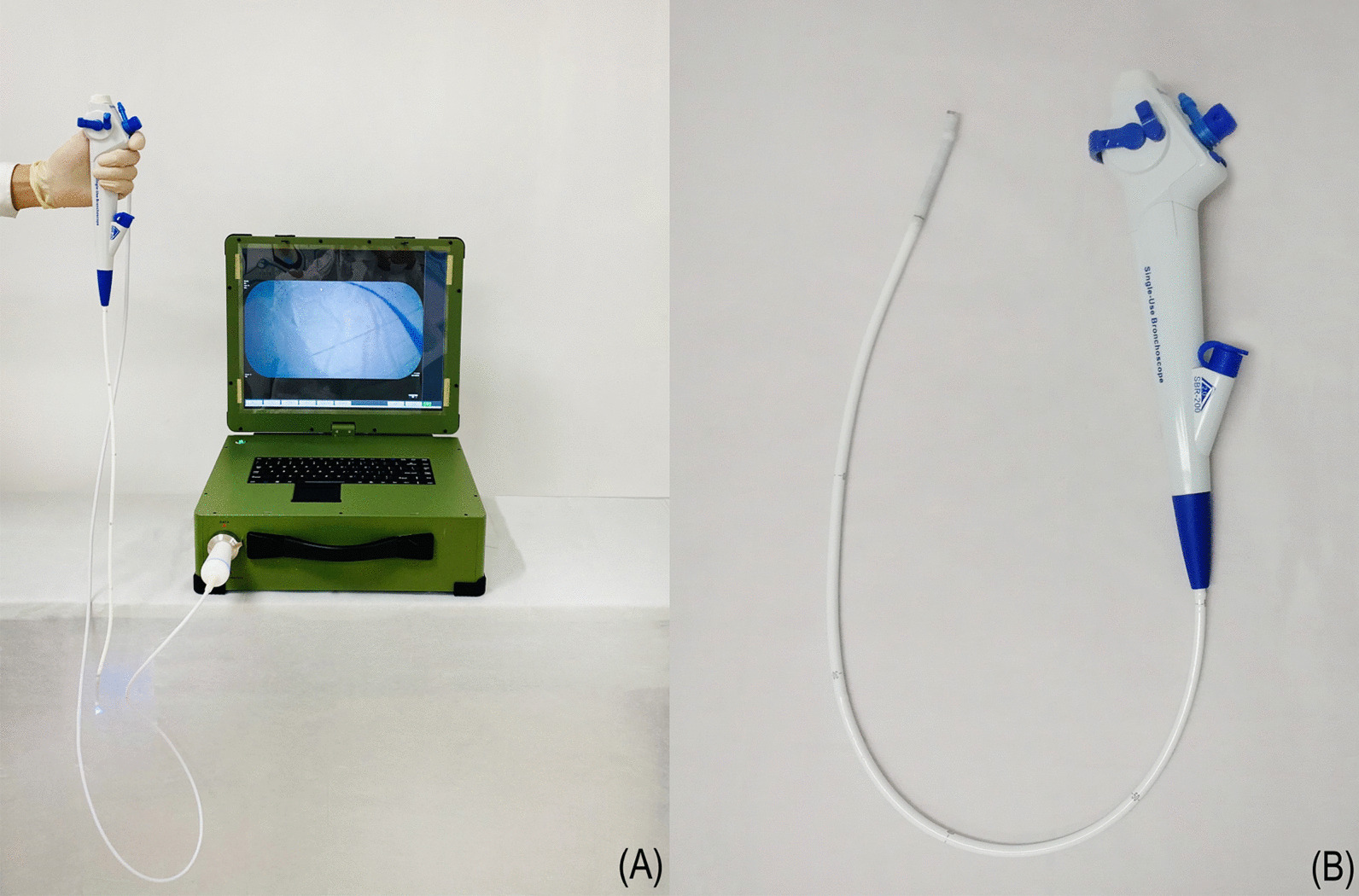
The YunSendo-R bronchoscope system includes a portable processor (**A**) and a disposable bronchoscope (**B**)

We developed the YunSendo-R bronchoscopy system (Fig. 1). It includes a disposable bronchoscope and a portable processor that can also connect to our previously developed gastrointestinal endoscope. The total weight of the system is 13.8 kg, and it can be powered by a built-in battery that lasts over 4 h or by an alternating current. External size of the processor is 400 mm × 450 mm × 160 mm (height is 560 mm in the unfolded position). The bronchoscope working length is 600 mm, and the scope shaft outer diameter is 5.5 mm with a 2.0 mm working channel. The scope tip allows flexion to 210° and retroflexion to 120°. In addition, it has 110 ± 10° field of view and 3–60 mm depth of field.

In this study, eight physicians used three types of bronchoscopes, the YunSendo-R bronchoscope (SBR-200; Huizhou Xzing, Guangdong, China), the Olympus bronchoscope (BF-H290; Olympus Medical Systems, Tokyo, Japan), and the Ambu bronchoscope (Ambu® aScope3™; AMBU, Copenhagen, Denmark), to operate on anaesthetised Guangxi Bama mini pigs. The order of bronchoscope was determined by drawing lots. The indexes used to assess the functions of the three types of bronchoscopes included image clarity, image colour contrast, image illumination, capture sensitivity, and field of view for visual ability evaluation, suction ability, working channel insertion, button sensitivity, and scope tip flexibility for manoeuvrability. Each item was scored using the 10-point Likert scale, where 1 corresponds to poor, 5 and 6 corresponds to neutral, and 10 corresponds to excellent (details in Table 1). The time was recorded, and operators were asked to complete the questionnaire after the bronchoscopy. These procedures were repeated three times on different pigs.

**Table 1:** Operator perception scores of three bronchoscope (Median (IQR))

		YunSendo-R	Olympus	Ambu
Image clarity	1: can't figure → 10: optimal clear	9 (8–9.25)	9 (9–10)	7 (5.75–7)*
Image color contrast	1: can't figure → 10: optimal sharp	9 (8–9.25)	9 (8.75–9.25)	7 (5.75–7.25)*
Image illumination	1: can’t figure → 10: optimal bright	9 (8–9.25)	9.5 (9–10)	7 (6.75–8)*
Capture sensitivity	1: no reaction → 10: instant	8 (8–8.25)	9 (8–9.25)	7 (7–8)
Field of view	1: narrowest → 10: broadest	8.5 (8–9.25)	9 (8.75–9.25)	8 (8–8)
Suction ability	1: can't suck → 10: optimal power	9 (8.75–9)	9 (9–10)	8 (7.75–8.25)
Working channel insertion	1: can't passage → 10: easy to passage	8.5 (7.75–9)	9.5 (9–10)	8 (7–8)
Button sensitivity	1: no reaction → 10: instant	8 (7.5–9)	9 (9–10)*	8 (7–9)
Scope tip flexibility	1: can't flex → 10: easy to flex	9.5 (8–10)	9 (8–9.25)	8 (7–8)*

^§^ IQR, interquartile ranges; YunSendo-R, YunSendo-R bronchoscope (SBR-200); Olympus, Olympus bronchoscope (BF-H290); Ambu, Ambu bronchoscope (Ambu®ascope3™)

*P < 0.05 comparing to YunSendo-R bronchoscope

Descriptive statistics are presented as mean ± standard deviation when normally distributed or as median (interquartile ranges) when non-normally distributed. Group comparisons were performed using the Wilcoxon rank-sum test or t-test (when appropriate). All statistical analyses were conducted using SPSS statistics version 21 (IBM, Armonk, New York, USA). *P*-values of < 0.05 were considered statistically significant.

## Results

**Fig. 2:**
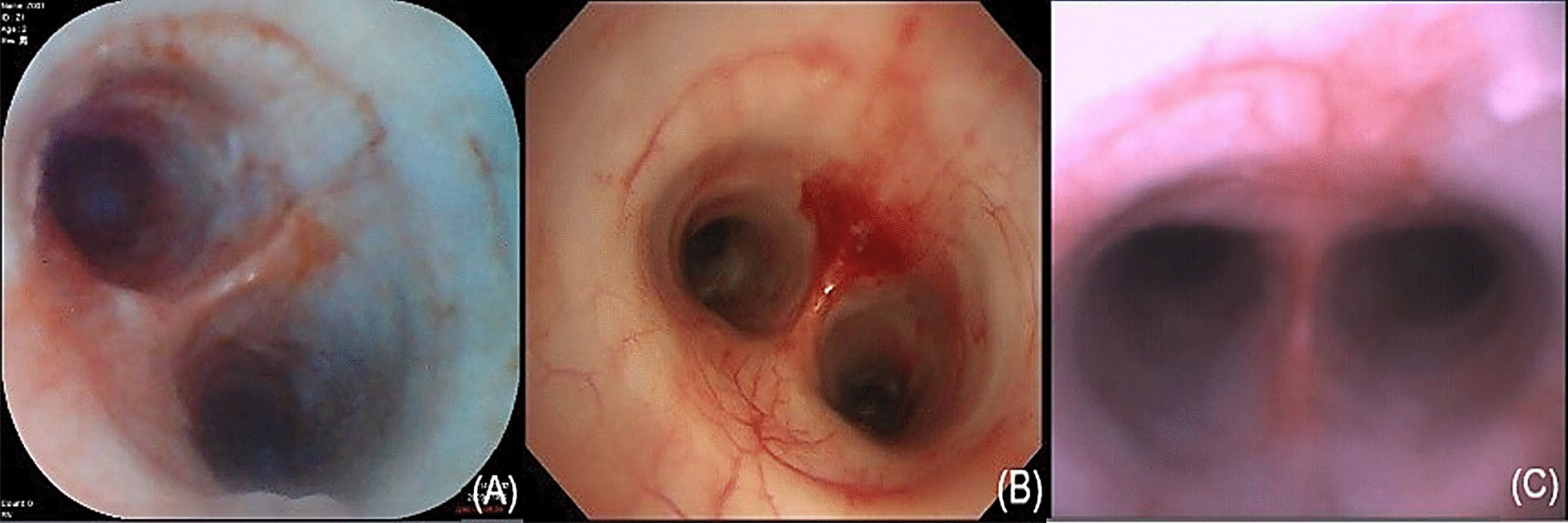
Three bronchoscope images of trachea carina. **A** YunSendo-R bronchoscope (SBR-200); **B** Olympus bronchoscope (BF-H290); **C** Ambu bronchoscope (Ambu®ascope3™)

**Fig. 3:**
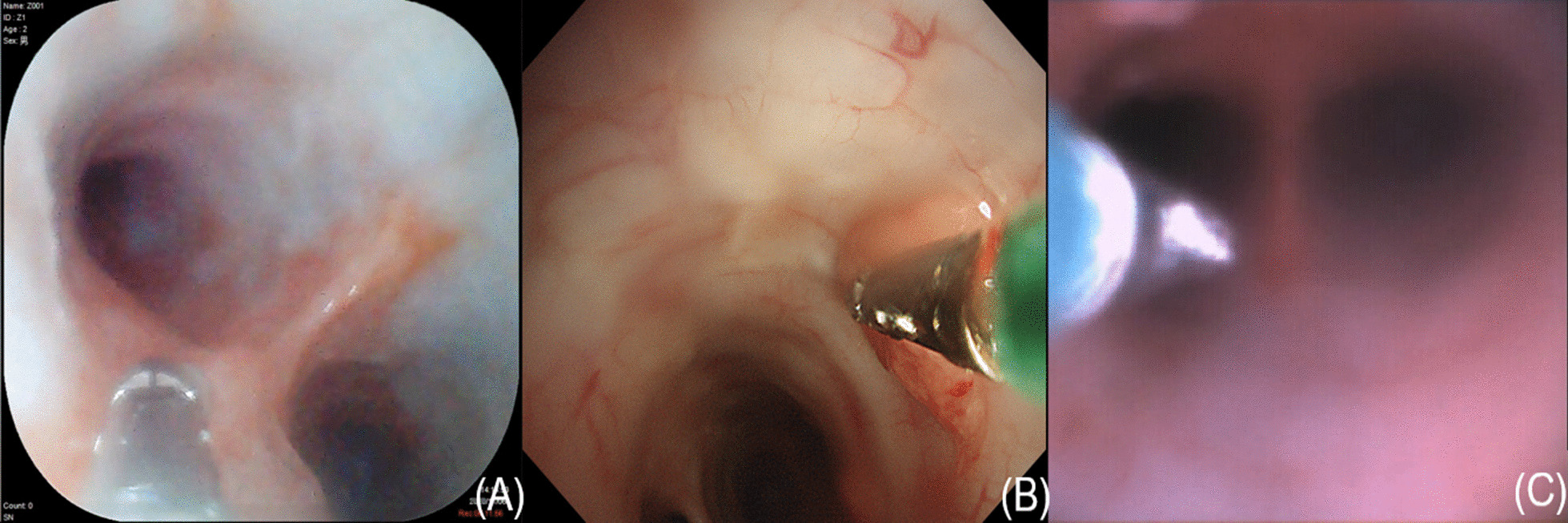
Three bronchoscope images of biopsy operation simulation. **A** YunSendo-R bronchoscope (SBR-200); **B** Olympus bronchoscope (BF-H290); **C** Ambu bronchoscope (Ambu®ascope3™)

All three types of bronchoscopes could be used to identify segmental bronchus and simulate biopsy operation successfully without any complications (Figs. 2, 3). The average operating times of the YunSendo-R bronchoscope, Olympus bronchoscope, and Ambu bronchoscope were 235.75 ± 56.29 s, 220.50 ± 42.00 s, and 225.88 ± 59.79 s, respectively. No statistical differences were found among the three bronchoscopes.

Operator perception scoring results are reported in Table 1. In visual ability assessment, the YunSendo-R bronchoscope showed no statistical difference with the Olympus bronchoscope (*P* > 0.05) and performed better than the Ambu bronchoscope in image clarity, colour contrast, and illumination assessments (*P* < 0.05). The YunSendo-R bronchoscope showed no statistical difference (*P* > 0.05) with the Ambu bronchoscope in capture sensitivity or field of view scores.

In terms of bronchoscope manoeuvrability, the score of the YunSendo-R bronchoscope was similar to the Olympus bronchoscope in suction ability, working channel insertion, and scope tip flexibility assessments (*P* > 0.05), while the YunSendo-R bronchoscope performed slightly worse than the Olympus bronchoscope in button sensitivity (*P* < 0.05). Compared to the Ambu bronchoscope, the YunSendo-R bronchoscope performed better in scope tip flexibility assessment (*P* < 0.05) and showed no statistical differences in the suction ability, working channel insertion, and button sensitivity score (*P* > 0.05). No complications were associated with all three bronchoscopes during the operation and until the 1-week follow-up.

## Discussion

The YunSendo-R bronchoscope system is a user-friendly system that can be mastered in a short period. Results showed that its operation time was not significantly different from that of the other two bronchoscopes. Moreover, the YunSendo-R bronchoscope images appeared to be of high quality, meeting the basic requirement for clinical bronchoscopy. In this study, no relevant complications such as perforation, bleeding, or pneumothorax were observed. This new system can be used to perform bronchoscopy effectively and safely.

Quality of images is a critical parameter during bronchoscopy as it has a direct and strong impact on the effectiveness of operation. Equipped with an advanced camera system including an integrated LED light and 12 megapixel lens, the YunSendo-R bronchoscope renders high-definition images to the monitor that are clear, sharp, and bright. There was no difference in the visual ability scores between the YunSendo-R and Olympus bronchoscope, which suggests that the YunSendo-R bronchoscope meets the basic clinical requirements in visual ability, similar to the reusable Olympus bronchoscope. Furthermore, the YunSendo-R bronchoscope performed better than the commercially available single-use Ambu bronchoscope on image clarity, colour contrast, and illumination assessments, indicating better perception of image for operators, which is conducive for the diagnosis and treatment of diseases.

**Fig. 4:**
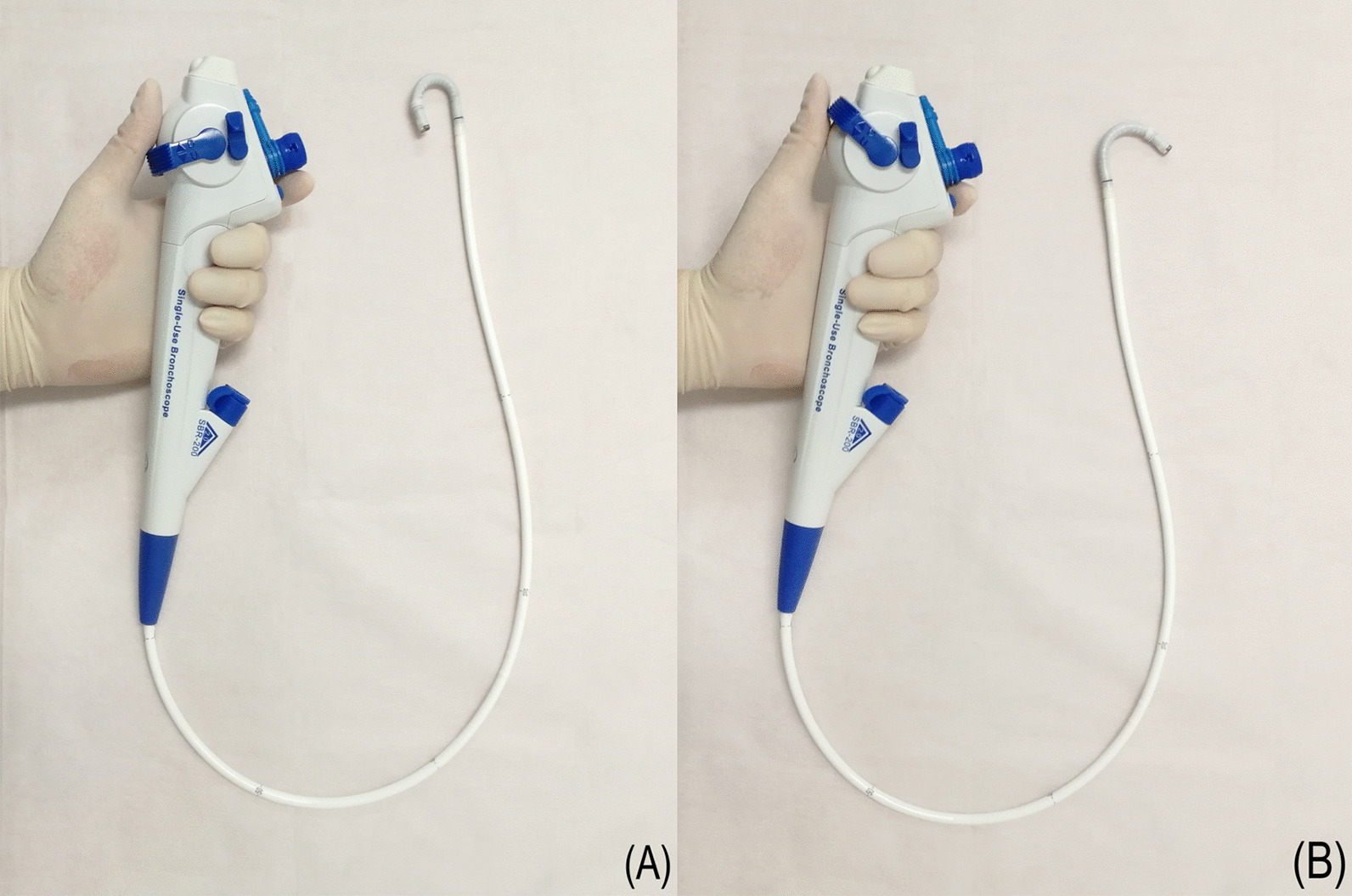
The YunSendo-R bronchoscope has a 210° up bending angles (**A**) and 120° down bending angles (**B**)

In terms of manoeuvrability evaluation, the YunSendo-R bronchoscope performed similar to the Olympus bronchoscope in suction ability, working channel insertion, and scope tip flexibility assessments, with the exception of button sensitivity where it requires further modification. Compared to the Ambu bronchoscope, the YunSendo-R bronchoscope had a higher score on the scope tip flexibility. The YunSendo-R bronchoscope has 210°/120° up/down bending angles (Fig. 4), making it easier to navigate into the segmental bronchus.

**Fig. 5:**
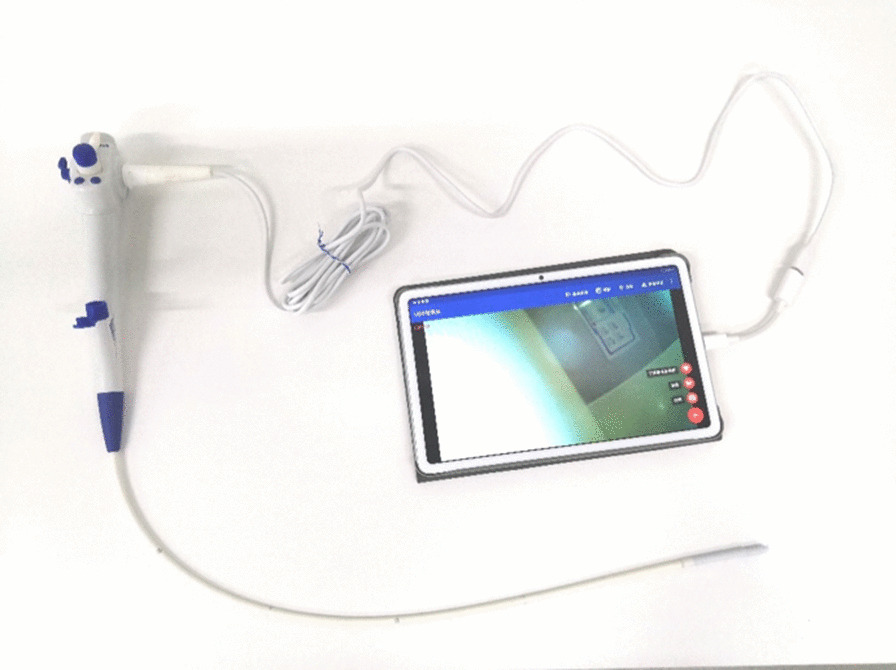
The YunSendo-R bronchoscope is connected with a small pad

The YunSendo-R bronchoscopic system has uniqueness of portability, disposability and expandability. Our results manifest that it performs similar in visual ability and manoeuvrability to Olympus BF-H290 bronchoscope system which is inconvenient to transport and disposal while YunSendo-R bronchoscope performs better in visual ability than portable and disposable Ambu bronchoscope. We also realize that there is a commercial available portable Olympus bronchoscope MAF, which has no disposability compared to Ambu bronchoscope and YunSendo-R bronchoscope. Under some circumstances, the YunSendo-R bronchoscope can be connected to a smaller pad (Fig. 5), similar to Ambu bronchoscope system, providing easier accessibility and simpler set-up procedure but lacking vacuum suction. After its use, the disposable bronchoscope is processed as normal medical waste, eliminating complicated reprocessing procedures. Patients will benefit from the sterile and single-use bronchoscope, while avoiding the risk of horizontal pathogen transmission and infection particularly during the COVID-19 pandemic [12]. It will also save around $129.6 annually for routine maintenance and repair as the infrastructure of a reusable bronchoscope is vulnerable to be impaired in daily use and cleaning [13]. The portable host has a built-in battery and negative pressure pump; therefore, the system can be used to perform bronchoscopy without any external power or pressure supply. Apart from the bronchoscope, the host can be connected to our previously developed disposable gastrointestinal endoscope, which saves both the space and cost for additional equipment [14]. The suitcase-like size of the system enables it to be transported easily and quickly, providing accessibility of the system in settings with limited medical resources, such as those that might be encountered during the COVID-19 pandemic.

Our study has several limitations. First, evaluation on the bronchoscope was conducted using an animal model rather than human subjects. Human bronchus tends to be complicated in comparison to the simple and clear bronchus of an animal; thus, our results may not reflect the actual quality of the YunSendo-R bronchoscopy in human. The eight bronchoscopists may be influenced in their evaluation by knowing which bronchoscope they used and the inadequate number of participants could also lead to a potential sampling bias requiring expanded sample size.

## Conclusion

In conclusion, considering the ongoing COVID-19 pandemic, we developed a promising bronchoscopy system with the advantages of portability and disposability. We tested the safety and efficacy of the YunSendo-R bronchoscope system in an animal model. The system has similar visual ability and manoeuvrability when compared to a commercially available reusable Olympus bronchoscope and performs better than a current single-use Ambu bronchoscope in terms of image quality and scope tip flexibility. Further studies on the YunSendo-R bronchoscope’s efficacy and safety in humans are needed.

## Data Availability

The datasets used during the current study are available from the corresponding author on reasonable request.
